# Appropriate use of antimicrobial prophylaxis: an observational study in 21 surgical wards

**DOI:** 10.1186/s12893-015-0048-7

**Published:** 2015-05-14

**Authors:** Marco Testa, Michela Stillo, Sebastian Giacomelli, Silvia Scoffone, Pier Angelo Argentero, Enzo Carlo Farina, Carla Maria Zotti

**Affiliations:** School of specialization in Hygiene and Preventive Medicine, Turin, Italy; Department of Public Health and Pediatrics, University of Turin, Turin, Italy; Infection Control Unit, Rivoli and Pinerolo Hospital, Turin, Italy; Department of General Surgery, City of Science and Health, Turin, Italy

**Keywords:** Antibiotic Prophylaxis, Chemoprevention, Surgery, Guideline, Flow-chart, Appropriateness

## Abstract

**Background:**

Surveillance of Surgical Site Infections (SSI) in 2010 found 39 % compliance with hospital guidelines in Piedmont (Italy). The aim of the study was to estimate the appropriate use of antimicrobial prophylaxis and compliance with hospitals guidelines in surgical wards.

**Methods:**

This survey study took place in 21 surgery wards of 4 public hospitals. Forms were completed by public health resident doctors together with a medical ward referent and infection control nurses. 15 consecutive surgical procedures were randomly chosen from each ward. A total of 320 cases were analyzed. The study period was from July 2012 to January 2013. Data were collected using a survey form. A final score variable from 0 to 4 was given to each case. The results were compared with hospital and international guidelines. Data were analyzed using Epi-Info software.

**Results:**

Of the 320 cases collected, 63 were excluded; of the remaining 257 cases, 56.4 % of the procedures were appropriate (score 4), 15.2 % were acceptable and 28.4 % were not acceptable. The study found an unjustified continuation of antimicrobial prophylaxis in 17.1 % of the 257 cases, an unjustified re-start of antimicrobial therapy in 9.7 % and a re-dosing omission in 7.8 %.

**Conclusions:**

The study demonstrated critical problems in antimicrobial prophylaxis management in surgical wards due to a lack of compliance between hospitals and national guidelines, a shortage of specific and updated recommendations for some surgical interventions and incorrect local specific procedures. Coordination between local and national recommendations, strengthening of evidence based decisions and continuous sharing of policy updates are needed.

## Background

The definition of “Surgical Site Infection” (SSI) was introduced in the medical vocabulary in 1992 as a replacement for the previous definition of “surgical wound infection”. NNIS (National Nosocomial Infection Study) – NHSN (National Healthcare Safety Network) recognize incisional and organ/space SSIs using standardized surveillance criteria [1].

Although they are potentially preventable, surgical site infections continue to occur frequently, demonstrating that the complete elimination of microbial risk is not possible. Although infection control strategies have been implemented, SSIs are a frequent cause of morbidity and mortality. Patients with SSIs more frequently require additional surgeries, re-hospitalization or ICU recovery, with an increased mortality risk.

European Countries use a surveillance system consisting of standardized protocols; methods of surveillance were fully integrated into The European Surveillance System (TESSy) in October 2010; the first report shows the results of SSIs surveillance in Europe from 2008 to 2009, as well as the results of analysis of trends from 2006 to 2009 [2].

Every year, a national report is produced on SSIs data from eleven Italian regions and one hundred hospitals; in 2010, the infection risk was 2.4 % for non-orthopedic interventions and 1.2 % for orthopedic interventions [3].

Piedmont (4,457,335 inhabitants; 337,160 surgical interventions with 99,535 Day Hospital) began surgical intervention surveillance in 2005 [4, 5]. Since 2008, Piedmont has participated in national and European surveillance with an annual report. The most recent analysis demonstrates comparable frequencies between Italian and European data on 9,500 interventions from 2008 to 2011.

Antimicrobial prophylaxis is a high efficacy control measure for SSIs that is defined as drug administration before surgical-field bacterial contamination [6–8].

Recommendations for appropriate surgical antimicrobial prophylaxis can be found in national and international guidelines [7, 9–13]. In Italy, each hospital produces its own protocol (hospital guidelines) that includes indications for the use of antimicrobial prophylaxis and therapy, in agreement with a hospital Infection Control Committee (ICC) composed of clinicians, pharmacists and microbiologists [14].

Beginning in 2008, Piedmont implemented an indicator system to evaluate the organization, surveillance, control and education activities relating to healthcare-associated infections; in 2010, an indicator of SSI control was introduced, including antimicrobial prophylaxis surveillance, which showed conformance in only 39 % of cases.

As demonstrated by recent publications, the SSIs issue has national and international relevance [15–18]. In 2006, the Surgical Care Improvement Project (SCIP) was established in the United States with the goal of reducing surgical complications by 25 % by 2010; three of the six SCIP performance indicators related to SSI prevention concerned antimicrobial prophylaxis administration: timing, antibiotic selection and duration [19, 20]. These three aspects seem to be critical components of successful antimicrobial prophylaxis in all available studies.

This study was conducted on a sample of interventions for estimating, by means of a flowchart, the appropriateness of the use of antibiotic prophylaxis and the compliance with hospital guidelines.

## Methods

The survey was conducted between July 2012 and January 2013 within the regional programme for HAI prevention and control, using data regarding patients who, at that time, were currently admitted in the wards included in the study. This observational descriptive study was intended for the surveillance of antibiotic prophylaxis use in surgical wards, as required from public hospitals by the Piedmont county government. Data were collected by the hospitals according to the regional plan of surveillance and control of healthcare associated infection set by the Regional public health directorate that yearly renew operative indications (Regione Piemonte. Direzione Sanitá. Settore prevenzione veterinaria: contact sanita.pubblica@regione.piemonte.it). The outcomes of the analysis, developed by the Regional committee on healthcare associated infection, were shared with the Regional public health directorate and with each hospital of the network trough reports and scientific publications.

Data were anonymously collected in collaboration with Hospitals referent after obtaining a study approval from each hospital Management Team.

Each patient admitted in one of the hospitals included in the study, signed the consent of personal data processing document. Hospital Management Teams involved are responsible for data processing and managing and agreed to the collection of data from medical records, carried out under the supervision of Hospital operators. The analysis was performed using data collected for institutional purposes and linked with a regional program of surveillance (Circular No.1950/2001 ‘Requisiti di minima per la prevenzione del rischio infettivo nelle strutture ospedaliere della Regione Piemonte’, available on line in the web site of the Italian National Centre for Disease Prevention and Control-CCM: http://www.ccm-network.it/documenti_Ccm/prg_area1/Inf_Oss/Normativa_reg/Piemonte_Prev_minima_strutt_osped_01.pdf) therefore ethics committee approval was not required.

A total of 21 surgical wards from four different hospitals (re-named A, B, C, and D) were analyzed for a period of 6 months (from July 2012 to January 2013); of the 21 wards, there were five urologic surgery, four general surgery, three vascular surgery, three orthopedic surgery, two plastic surgery, one heart surgery, one thoracic surgery, one neurosurgery, and one otolaryngology ward. For each ward, data from almost 15 consecutive interventions randomly chosen were collected, for a total of 320 cases.

A flowchart-form was used to collect data to simplify, standardize and objectify the data collection process. Forms were filled in by a team composed of public health resident doctors together with a medical ward referent and an infection control nurse (ICN). A final score variable from 0 to 4 was given to each case. Hospital guidelines, generally based on 2008 national guidelines and approved by ICC, were used as the standard reference.

The flow chart-form is divided into four sections.

**Fig. 1:**
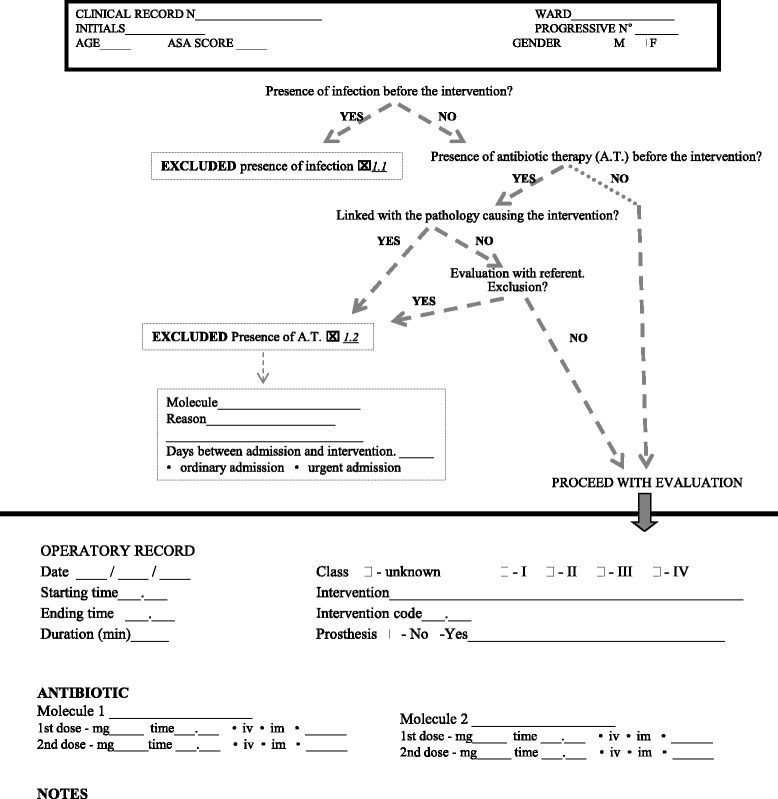
Flow chart. First part
- The second section (Fig. 2) is a flow chart including information about the appropriateness of the antimicrobial prophylaxis.

- The first section (Fig. 1) contains personal data (age, sex, number of medical records) and intervention data (date, contamination classification, International Classification of Diseases ICD9 code, prosthesis, duration and antibiotic use). In this section, interventions could be excluded due to infection or antibiotic therapy.

**Fig. 2:**
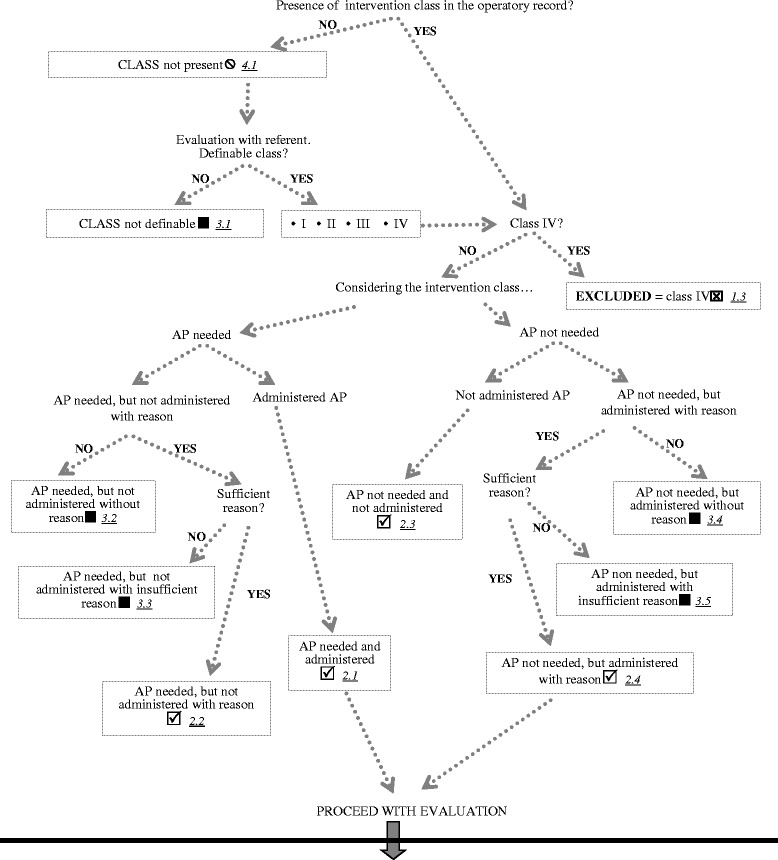
Flow chart. Second part
- The third section (Fig. 3) examines specific aspects of the antimicrobial prophylaxis: molecule, timing of first administration, re-dosing, last administration, modality and motivation for post-intervention antibiotic re-take.

**Fig. 3:**
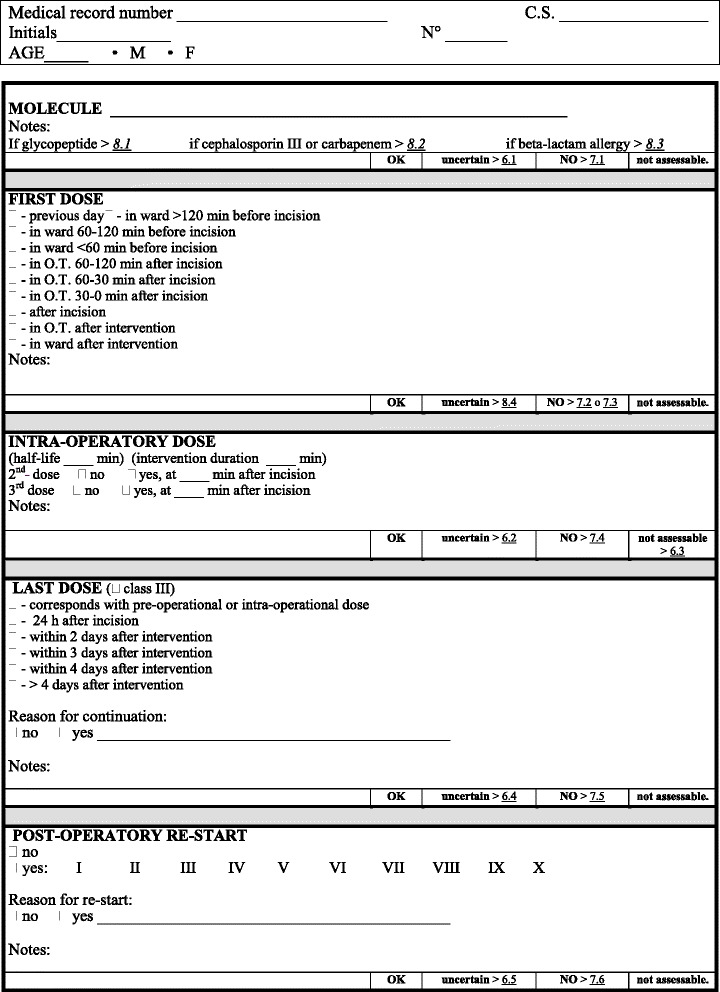
Antibiotic prophylaxis additional information

Each of these criteria contributed to produce a final score from 0 to 4.

**Fig. 4:**
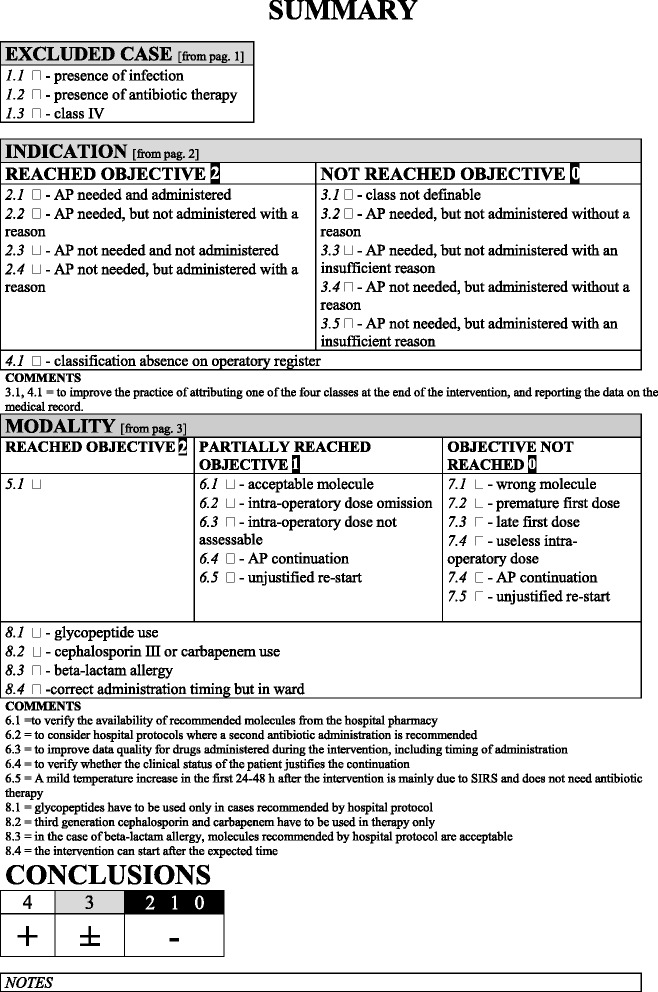
Antibiotic prophylaxis summary information

- The last section (Fig. 4) is a summary of the collected data. This part allows the researcher to give a final score to the appropriateness of the antimicrobial prophylaxis. Final scores are divided into three categories: 0–2: not conforming with antimicrobial prophylaxis; three: acceptable antimicrobial prophylaxis; four: completely correct antimicrobial prophylaxis.

Data were analyzed using the Epi-Info statistical software.

## Results and discussion

During the study, 320 clinical records were analyzed. Of these, 25 records were auto-compiled by ward personal and were excluded. Of the 295 remaining cases, those with infection or antimicrobial therapy before the intervention and those classified as class IV were excluded, with 257 cases remaining. Characteristics of patients and surgical procedures are reported in Table 1.

**Table 1:** Patient and surgical procedure characteristic

	Number	Percent
Gender		
- Missing ^a^	1	0.4
- Male	155	60.3
- Female	101	39.3
Age, years		
- Mean age	63.0 (44.1-81.9)	-
- Age range	9-98	-
ASA Score		
- Missing	69	26.8
- 1: in good general condition	29	11.3
- 2: patients with mild systemic disease	77	30.0
- 3: patients with moderate systemic disease and functional limitations	70	27.2
- 4: patients with serious systemic disease	12	4.7
- 5: patients with very serious condition with <24 h life expectancy, with or without surgical intervention	0	0.0
Surgical wound classification		
- Missing	3	1.2
- I (clean)	180	70.0
- II (clean/contaminated)	62	24.1
- III (contaminated)	12	4.7
- IV (dirty/infected)	Excluded	-
Implant of prosthesis		
- Missing	3	1.2
- Yes	109	42.4
- No	145	56.4
Antibiotic prophylaxis needed		
- Yes	217	84.4
- No	40	15.6

^a^We consider as missing all values not recorded on the medical chart

Hospital A procured 108 cases, hospital B 61 cases, hospital C 75 cases, and hospital D 51 cases. The study analyzed different types of interventions by different surgical disciplines. Urological and general surgery interventions constituted approximately 45 % of the sample; orthopedic and vascular surgery interventions each constituted 15 % of cases; and 20 % of interventions were divided among otolaryngology, neurosurgery, heart surgery and plastic surgery (Table 2).

**Table 2:** Interventions by hospitals

Surgical ward	Hospital A	Hospital B	Hospital C	Hospital D	Number	Percent
Urologic (surgery)	32	15	15	14	76	25.76
General (surgery)	15	16	15	15	61	20.68
Orthopedic (surgery)	-	15	15	17	47	15.94
Vascular (surgery)	31	-	15	-	46	15.60
Plastic (surgery)	15	-	-	5	20	6.78
Otolaryngologic (surgery)	-	15	-	-	15	5.08
Neuro (surgery)	-	-	15	-	15	5.08
Heart (surgery)	15	-	-	-	15	5.08
Total^a^	108	61	75	51	295	100

^a^Due to mathematical approximation the algebraic sum of % values is 100.1. In Table 2 the value Total % has been corrected to 100

The final score analysis showed that 56.4 % of procedures were completely correct, 15.2 % were acceptable and 28.4 % were non-conforming. However, the percent of procedures that were correct varied between the four hospitals, from 43.8 % as the minimum in hospital A to the maximum of 77.6 % in hospital B.

The analysis was performed by ward type; there was significant variation from a minimum of 32.6 % in vascular surgery to a maximum of 100.0 % in heart surgery (Table 3). The stratification using the American Society of Anesthesiologists score (ASA score) and the intervention class did not allow for the observation of significant differences in conformance. Errors included antimicrobial therapy continuation for longer than the time limits suggested by hospital protocols (17.1 %), antimicrobial therapy re-starting without valid justification (9.7 %), re-dosing omission (7.8 %), molecule not indicated (7.4 %) and antimicrobial prophylaxis use in interventions without protocol indications (6.2 %) (Table 4).

**Table 3:** Final scores for appropriateness of antibiotic prophylaxis by surgical specialty

Surgical ward	≤2 (not conforming)		3 (acceptable)		4 (correct)		Total
	N	%	N	%	N	%	N
Urologic (surgery)	21	30.9	14	20.6	33	48.5	68
General (surgery)	13	27.1	12	25.0	23	47.9	48
Vascular (surgery)	22	51.2	7	16.3	14	32.6	43
Orthopedic (surgery)	2	4.7	4	9.3	37	86.0	43
Plastic (surgery)	6	40.0	1	6.7	8	53.3	15
Heart (surgery)	0	0.0	0	0.0	15	100	15
Otolaryngologic (surgery)	5	38.5	0	0.0	8	61.5	13
Neuro (surgery)	4	33.3	1	8.3	7	58.3	12
Total (all interventions)	73	28.4	39	15.2	145	56.4	257

**Table 4:** Frequency of antimicrobial prophylaxis-linked “errors” ^a^

Type of error	Number of errors^a^	Percent
Antimicrobial prophylaxis continuation	44	17.1
Antibiotic therapy re-start	25	9.7
Intra-operatory dose omission	20	7.8
“Wrong” molecule	19	7.4
Antimicrobial prophylaxis administered (without a reason) when not needed	16	6.2
Antimicrobial prophylaxis not administered (without a reason) when needed	7	2.7
Antimicrobial prophylaxis not administered (with insufficient reason) when needed	1	0.4
Un-necessary intra-operatory dose	0	0.0
Premature first dose administration	1	0.4
Late first dose administration	0	0.0
Total^b^	133/257	51.8

“Wrong”: molecule is not included in hospital guidelines

^a^Due to the high heterogeneity of antibiotic prophylaxis guide line published in recent years, and compatibly with the aim of the study, specific ward antibiotic prophylaxis protocols were accepted only when proven to be evidence based and if approved by ICC

^b^Total interventions = 257; multiple errors are possible for each intervention

Frequent errors were analyzed to understand problems characterizing different surgical areas, with results stratified by surgical specialty. More variability was observed in general surgery, urologic and vascular surgery, where mistakes are concentrated in certain areas: antimicrobial prophylaxis continuation (22.5 %) or antimicrobial therapy retake (25 %) after the intervention in urologic surgery, intra-operatory dose omission in general surgery (18.8 %), incongruous continuation (30.2 %) and wrong molecule in vascular surgery (20.9 %) (Table 5). An analysis of 44 interventions with unjustified antimicrobial prophylaxis continuation found that in 70.5 % of cases the drug was administered for more than 24 h and in 50 % of cases for more than 48 h.

**Table 5:** Five most frequent errors by surgical specialty

Errors	Urologic surgery		General surgery		Vascular surgery		Orthopedic surgery		Plastic surgery		Heart surgery		ORL		Neuro surgery		Total	
N	68		48		43		43		15		15		13		12		257	
	n	%	n	%	n	%	n	%	n	%	n	%	n	%	n	%	n	%
Antimicrobial prophylaxis continuation	15	22.5	7	14.6	13	30.2	2	4.6	4	26.7	0	0.0	1	7.7	2	16.7	44	17.1
Antibiotic therapy re-start	17	25.0	2	4.2	1	2.3	1	2.3	4	26.7	0	0.0	0	0.0	0	0.0	25	9.7
Intra-operatory dose omission	4	5.9	9	18.8	4	9.3	0	0.0	3	20.0	0	0.0	0	0.0	0	0.0	20	7.8
“Wrong” molecule	4	5.9	2	4.2	9	20.9	0	0.0	1	6.7	0	0.0	2	15.4	1	8.3	19	7.4
Antimicrobial prophylaxis administered (without a reason) when not needed	1	1.5	4	8.3	7	16.3	0	0.0	1	6.7	0	0.0	3	23.1	0	0.0	16	6.2

“Wrong”: molecule is not included in hospital guidelines

The most common errors were studied using as the denominator only the interventions for which that type of error was possible; therefore, in Table 6 the frequencies are more elevated; for example, the error of re-dosing occurred in 90.9 % of cases.

**Table 6:** “Real” frequencies of the most frequent errors by surgical specialty

Errors	Urologic surgery		General surgery		Vascular surgery		Orthopedic surgery		Plastic surgery		Heart surgery		ORL		Neuro surgery		Total	
	n	%	n	%	n	%	n	%	n	%	n	%	n	%	n	%	n	%
Antimicrobial prophylaxis continuation	15/37	40.5	7/19	36.8	13/21	61.9	2/16	12.5	4/4	100	0/15	0	1/5	20.0	2/9	22.2	44/126	34.9
Antibiotic therapy re-start	17/25	68.0	2/5	40.0	1/3	33.3	1/6	16.7	4/4	100	0/0	-	0/0	-	0/5	0	25/48	52.1
Intra-operatory dose omission	4/4	100	9/10	90.0	4/4	100	0/0	-	3/3	100	0	0	0/0	-	0/1	0	20/22	90.9
“Wrong” molecule	4/62	6.5	2/37	5.4	9/31	29.0	0/42	-	1/20	5.0	0/12	-	2/6	33.0	1/10	10.0	19/213	8.9
Antimicrobial prophylaxis administered (without a reason) when not needed	1/5	20.0	4/11	36.4	7/10	70.0	0/1	0.0	1/5	20.0	0/0	-	3/7	42.9	0/1	0	16/40	40.0

“Wrong”: molecule not included in hospital guidelines

Antimicrobial prophylaxis is important because it represents approximately 40–50 % of total hospital-prescribed antibiotics [21]. Indiscriminate antibiotic use increases the prevalence of antibiotic-resistant bacteria and predisposes patients to infections, such as *Clostridium difficile colitis* [22, 23]. Clinical and efficacy studies on antimicrobial prophylaxis have identified antibiotic choice, timing, intra-operatory re-dosing and duration as the key points to ensure SSI prevention.

Our analysis found that antimicrobial prophylaxis did not conform to guidelines in 28.4 % of cases; in contrast, antimicrobial prophylaxis was correct in 56.4 % of cases. This value is comparable to data in the literature that show an adherence to guidelines of 48 % to 70.7 % [17, 24].

Not-uniformity in clinical record compilation, depending on hospital and ward, interfered with data collection. Consultation with the ward referent helped with clarification, in particular about antimicrobial prophylaxis timing and continuation; the presence of the ward referent was crucial for doubtful cases because of their direct patient knowledge. It is important to standardize decisions about antimicrobial prophylaxis; these types of process evaluations should not require an interpretation of prescribing behavior.

Two of the major limitations of the flowchart were:

- The flowchart lacks the ability to evaluate the appropriate use of antibiotics that are not included in hospital guidelines, but that have a compatible spectrum (i.e., amoxicillin plus clavulanic acid instead of cefazolin).
- In some cases, it was difficult to distinguish between antimicrobial prophylaxis continuation and an antibiotic therapy re-start. Heart surgery was the only category to obtain a final score of 100 % correct; because of an elevated Methicillin-Resistant *Staphylococcus Aureus* (MRSA) isolation frequency, the heart surgery unit had an internal protocol (including vancomycin + cefazoline) that was validated by ICC and compliant with hospital and national guidelines.

The national guidelines lack indications about antimicrobial prophylaxis in plastic surgery interventions; in one plastic surgery ward, an internal protocol was introduced that was not validated by ICC and not compliant with recent indications from the literature [25, 26].

The comparison between hospital and national guidelines showed some differences that might have influenced the final scores of some interventions:

1. *Use of glycopeptide*: National guidelines recommend the use of glycopeptides only in the case of MRSA isolation that is greater than 30 %. Hospital C guidelines allow the administration of vancomycin for patients coming from long-term care or for patients who had been admitted to a hospital ward 1 week before the intervention. This protocol, which was established based on recommendations from specialists, was based on evidence that patients with those features are more likely to be colonized by MRSA. In Hospital C, the isolation of MRSA in surgical wards was found to be approximately 50 %.
2. *Use of piperacillin + tazobactam*: National guidelines limit the use of this type of antibiotic to therapy, whereas the internal protocol of Hospital C recommends their use in some specific urological and general surgery interventions involving the intestine.
3. *Prophylaxis duration*: National guidelines recommend a singular administration of antibiotic, other than for interventions with a high risk of contamination. In the guidelines of Hospital D, the extension of the prophylaxis in the first 24 h is recommended for several interventions (general surgery, urological surgery, vascular surgery, neurosurgery), without considering the risk of contamination. Breast surgery is another example of this problem: national guidelines recommend a singular administration of cefazolin in ASA ≥3 patients, with the possibility of continuation for 24 h. Based on these recommendations, Hospital D guidelines suggest three administrations of cefazolin in the first 24 h for breast interventions.

Such a feature is extensively discussed in the scientific literature; however, a clear and shared recommendation still needs to be developed.

Recent remarks note problems with the extension of prophylaxis and more studies are necessary to prove a real improvement from prolonging antimicrobial prophylaxis for 24 h, in terms of surgical site infection risk and health-care costs [13].

## Conclusion

This study shows a variation in compliance with national and hospital guidelines; moreover, the lack of specific recommendations for some interventions increases the use of practices that are based on surgeon experiences and are not always evidence based.

The study provided an opportunity to implement a standardized instrument to collect data, with the aim of obtaining an objective evaluation. The flowchart enabled comparisons across different settings and in different operative conditions. Even if the flowchart may be improved, it was found to be a good operative tool.

Some common behaviors (unjustified antimicrobial prophylaxis continuation, inappropriate antibiotic therapy re-take, missed re-dosing, wrong molecules and unjustified antimicrobial prophylaxis) indicate the need for a scientific debate about controversial antimicrobial prophylaxis features; training activities and audit techniques are tools to address the question and obtain shared and evidence-based recommendations.

The alignment of local policies with national recommendations seems to be required; some differences between local protocols and national guidelines might be caused by missing updates in guidelines.

Medical choices should be evidence-based, aiming both to improve health-care quality and to reduce health-related costs.

## Abbreviations

SSI: Surgical Site Infections
NNIS: National Nosocomial Infection Study
NHSN: National Healthcare Safety
TESSy: The European Surveillance System
ICC: Infection Control Committee
SCIP: Surgical Care Improvement Project
ICN: Infection Control Nurse
ICD9: International Classification of Diseases
ASA score: American Society of Anesthesiologists score
MRSA: Methicillin-Resistant *Staphylococcus Aureus*

## References

[1] Horan TC, Gaynes RP, Martone WJ, Jarvis WR, Emori TG (1992). CDC definitions of nosocomial surgical site infections, 1992: a modification of CDC definitions of surgical wound infections. Infect Control Hosp Epidemiol.

[2] European Centre for Disease Prevention and Control (2012). Surveillance of surgical site infections in Europe 2008–2009.

[3] ASSR Emilia Romagna (2012). Sorveglianza delle infezioni del sito chirurgico in Italia. Interventi ortopedici anno 2010. Interventi non ortopedici anno 2011.

[4] Castella A, Argentero PA, Farina EC, Anselmo E, Djiomo A, Zotti CM (2009). Surgical site infections surveillance in Northern Italy. Infection.

[5] Castella A, Argentero PA, Farina EC, Charrier L, Del Prever EM, Zotti CM (2010). Incidence of surgical-site infections in orthopaedic surgery: a Northern Italian experience. Epidemiol Infect.

[6] Mangram AJ, Horan TC, Pearson ML, Silver LC, Jarvis WR (1999). Guideline for prevention of surgical site infection. Infect Control Hosp Epidemiol.

[7] National Institute for Health and Clinical Excellence: Surgical site infection. Prevention and treatment of surgical site infection. 2008. https://www.nice.org.uk/guidance/cg74. Accessed 8 May 2015.

[8] De Lalla F (1999). Chemioantibioticoprofilassi in chirurgia.

[9] Istituto Superiore di Sanità: Sistema Nazionale per le Linee Guida - Antibioticoprofilassi perioperatoria nell’adulto. 2008. http://www.snlg-iss.it/lgn_antibioticoprofilassi_perioperatoria_adulto_2008. Accessed 8 May 2015.

[10] ASSR Emilia Romagna (2010). Compendio delle principali misure per la prevenzione e il controllo delle infezioni correlate all’assistenza.

[11] Network SIG (2008). Antibiotic prophylaxis in surgery. A national clinical guideline.

[12] Institute For Clinical Systems Improvement (2009). Antibiotic prophylaxis for surgical site infection prevention in adults.

[13] Bratzler DW, Dellinger EP, Olsen KM, Perl TM, Auwaerter PG, Bolon MK (2013). Clinical practice guidelines for antimicrobial prophylaxis in surgery. Am J Health-Syst Pharm.

[14] Moro ML, Marchi M, Buttazzi R, Nascetti S, INF-OSS Project Group (2011). Progress in infection prevention and control in Italy: a nationwide survey. J Hosp Infect.

[15] Prospero E, Barbadoro P, Marigliano A, Martini E, D’Errico MM (2011). Perioperative antibiotic prophylaxis: improved compliance and impact on infection rates. Epidemiol Infect.

[16] Pittalis S, Ferraro F, Piselli P, Ruscitti LE, Grilli E, Lanini S (2013). Appropriateness of surgical antimicrobial prophylaxis in the Latium Region of Italy, 2008: a multicenter study. Surg Infect (Larchmt).

[17] Friedman ND, Styles K, Gray AM, Low J, Athan E (2013). Compliance with surgical antibiotic prophylaxis at an Australian teaching hospital. AJIC.

[18] Hohmann C, Eickhoff C, Radziwill R, Schulz M (2012). Adherence to guidelines for antibiotic prophylaxis in surgery patients in German hospitals: a multicentre evaluation involving pharmacy interns. Infection.

[19] Bratzler W, Hunt DR (2006). The surgical infection prevention and surgical care improvement projects: national initiatives to improve outcomes for patients having surgery. Clin Infect Dis.

[20] Napolitano LM (2010). Perspectives in surgical infections: what does the future hold?. Surg Infect (Larchmt).

[21] Moss F, McNicol MW, McSwiggan DA, Miller DL (1981). Survey of antibiotic prescribing in a district general hospital. I. Pattern of use. Lancet.

[22] Goossens H, Ferech M, Vander Stichele R, Elseviers M, ESAC Project Group (2005). Outpatient antibiotic use in Europe and association with resistance: a crossnational database study. Lancet.

[23] Jobe BA, Grasley A, Deveney KE, Sheppard BC (1995). Clostridium difficile colitis: an increasing hospital-acquired illness. Am J Surg.

[24] Hawkins RB, Levy SM, Senter CE, Zhao JY, Doody K, Kao LS (2013). Beyond surgical care improvement program compliance: antibiotic prophylaxis implementation gaps. Am J Surg.

[25] Toia F, D’Arpa S, Massenti MF, Amodio E, Pirrello R, Moschella F (2012). Perioperative antibiotic prophylaxis in plastic surgery: a prospective study of 1100 adult patients. J Plast Reconstr Aesthet Surg.

[26] Hunter JG (2007). Appropriate prophylactic antibiotic Use in plastic surgery: the time has come. Plast Reconstr Surg.

